# Manual of Anatomy

**Published:** 1907-12

**Authors:** 


**Manual of Anatomy.** By A. M. Buchanan, M.A., M.D. Vol. II. Pp. xv., 575—1539. London: Baillière, Tindall & Cox. 1907.

It is impossible to review in any detail a book of over 1,500 closely printed pages in the space at our disposal. This volume deals with the abdomen, head and neck, and thorax, and is published in the same style and in series with Rose and Carless's *Surgery*. It forms a much less cumbrous book than any of the single-volume anatomies, and is printed clearly and illustrated well. The regional method of description is adopted, which, together with some directions for dissection, makes the book a manual for the dissecting-room as well as a systematic treatise. There can be no doubt that the whole work being by the same hand is an advantage far greater than any obtained by the system of multiple authors in such a subject as anatomy. The letterpress and descriptions are extremely clear and accurate ; but it is to be regretted that not only are the well-known personal names, such as Scarpa's or Cotle's fascia, retained, but many others which are new to English students are introduced, such as Houston's muscle for the anterior part of the bulbo-cavernosus. and the muscle of Guthrie for the constrictor urethræ. This involves an unnecessary effort of memory in a subject which already taxes that faculty to the utmost.

The manner in which the embryology and histology of each organ are described after the gross anatomy is completed is a most excellent plan, and one which almost compels the student's attention, whereas separate chapters of these subjects are apt to be overlooked or forgotten. The illustrations of the abdomen, thorax, and nervous system are very clear, and just fulfil the purpose for which they are intended. But many of the pictures of the head and neck are far too small and complicated, and would necessitate a reference to larger text-books or atlases. Taken as a whole, the book is a fresh and clear exposition of an intricate subject, and it will probably be welcomed for its conciseness and become as popular among students as the other members of the same series of manuals.

